# An Integrated Surgical Management for Giant Dermatofibrosarcoma Protuberans of Anterior Abdominal Wall

**DOI:** 10.7759/cureus.17038

**Published:** 2021-08-09

**Authors:** Swagata Brahmachari, Anubha Pandey, Mahendra Pratap Singh, Vandana Agarwal

**Affiliations:** 1 Department of General Surgery, All India Institute of Medical Sciences, Bhopal, IND; 2 Department of Pathology, Atal Bihari Vajpayee Government Medical College, Vidisha, IND; 3 Department of Pathology, LN Medical College and Research Centre, Bhopal, IND

**Keywords:** synthetic mesh, dermatofibrosarcoma protuberans, giant, three-dimensional excision, abdominal wall reconstruction, component separation technique

## Abstract

Giant dermatofibrosarcoma protuberans (DFSP) is a very rare dermal sarcoma whose diagnosis and management are important because of the high local recurrence but low metastatic potential. Complete surgical excision of giant DFSP in a single stage is difficult but has a high cure rate.

A 47-year-old man presented with a gradually increasing large (18 x 15 x 7 cm) DFSP in the epigastrium. A 3 cm circumferential wide local excision (WLE) with microscopic tumor-free margin confirmed by frozen section was performed. Immediate single staged tension-free primary closure of resultant defect was done on the principle of abdominal wall reconstruction (AWR) in ventral hernia repair. This technique of anterior component separation and bridge meshplasty is functional, avoids multiple surgeries, is cost-effective, and can be done in a resource-limited setting in developing countries.

A multidisciplinary and integrated surgical approach to treat giant DFSP over epigastrium, by three-dimensional WLE and immediate AWR with anterior component separation technique (CST) and bridging meshplasty, can be of immense help in managing such rare cases in developing countries.

## Introduction

Dermatofibrosarcoma protuberans (DFSP) are rare, cutaneous mesenchymal neoplasm with an annual global incidence of 0.8-4.5 cases per million persons. Hoffman named this rare clinical entity DFSP which accounts for 1-6% of soft tissue sarcomas and 18% of cutaneous soft tissue sarcomas [1]. DFSP is a superficial, slow-growing, locally aggressive, infiltrative cutaneous sarcoma arising from the dermis. It is composed of CD-34 positive, neoplastic spindle cells with high local recurrence and low metastatic potential [2]. Large-sized DFSP and delayed presentation are often due to rarity and misdiagnosis as a benign tumor because of its indolent evolution. Usually, it presents as a 1-6 cm-sized tumor, but rarely >20 cm have also been reported [2]. Clinical awareness of this uncommon oncological entity is important as complete surgical excision with histologically negative margin has an excellent prognosis with a 99% five-year survival rate [3]. Repair of the resultant defect in the anterior abdominal wall is necessary to maintain function. So we present an integrated surgical approach to treat a giant DFSP over epigastrium by three-dimensional wide local excision (WLE) and immediate abdominal wall reconstruction (AWR) by anterior component separation technique (CST) with bridging meshplasty.

## Case presentation

**Figure 1:**
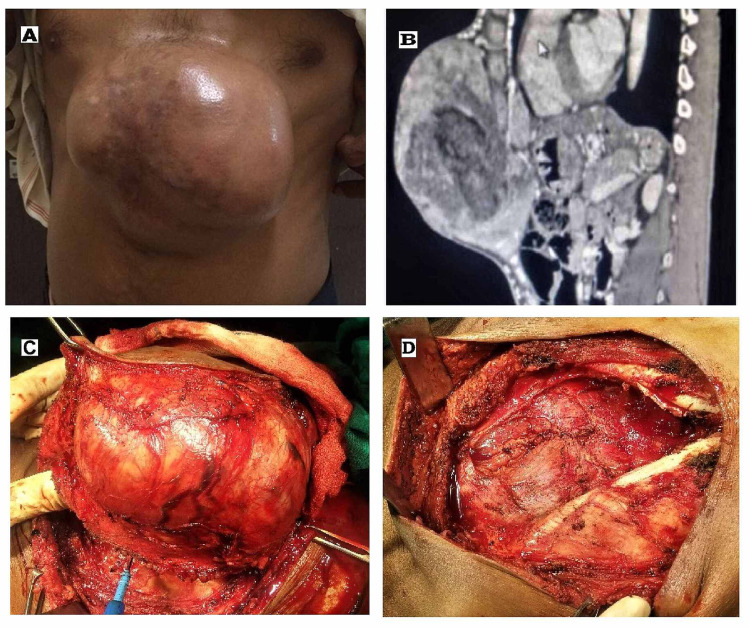
Gross and perioperative view of dermatofibrosarcoma protuberans. 1(A):  A large protuberant mass located in the epigastrium. 1(B): MRI (chest and abdomen) showing large heterogenous mass (16.4 x 14.6 x 9.7 cm) on the anterior abdominal wall in the epigastric region, arising from rectus abdominis muscle, and the gastric antrum appears adherent to the deeper margin of the lesion. 1(C): Three-dimensional wide local excision being done with 3 cm macroscopic clear margin. 1(D): Resultant full-thickness abdominal wall defect (24 x 21 cm) following en-block excision of tumor in the epigastrium and the peritoneal defect repaired.

A 47-year-old man presented with a slow-growing large tumor in the epigastrium for four years with occasional pain. Clinically, a 15 x 14 cm large, lobulated, ﬁrm, non-tender tumor covered by shiny telangiectatic skin was present over the anterior abdominal wall of the epigastrium (Figure 1A). Radio imaging studies (contrast-enhanced computed tomography [CECT], MRI) revealed a large, heterogeneous, exophytic tumor measuring 16.4 x 14.6 x 9.7 cm over the anterior abdominal wall of epigastrium, adherent to skin, subcutaneous tissue, and underneath rectus muscle in the midline, without any visceral metastasis or regional lymphadenopathy (Figure 1B). Complete surgical excision with immediate reconstruction was planned by an integrated multidisciplinary team, as the patient was unable to afford neoadjuvant imatinib therapy. A three-dimensional excision of the tumor of size 18 x 15 x 10 cm, with a three-centimeter macroscopic tumor-free margin circumferentially, including underlying rectus sheath and peritoneum, was performed (Figure 1C). Frozen section evaluation showed negative microscopic peripheral and deep margin. Further, one cm shaved peripheral margin along with the en-blocked excised tumor was sent for histopathological evaluation. The resultant full-thickness abdominal wall defect of size 22 x 19 cm (Figure 1D) was reconstructed by modified anterior CST with bridging meshplasty.

**Figure 2:**
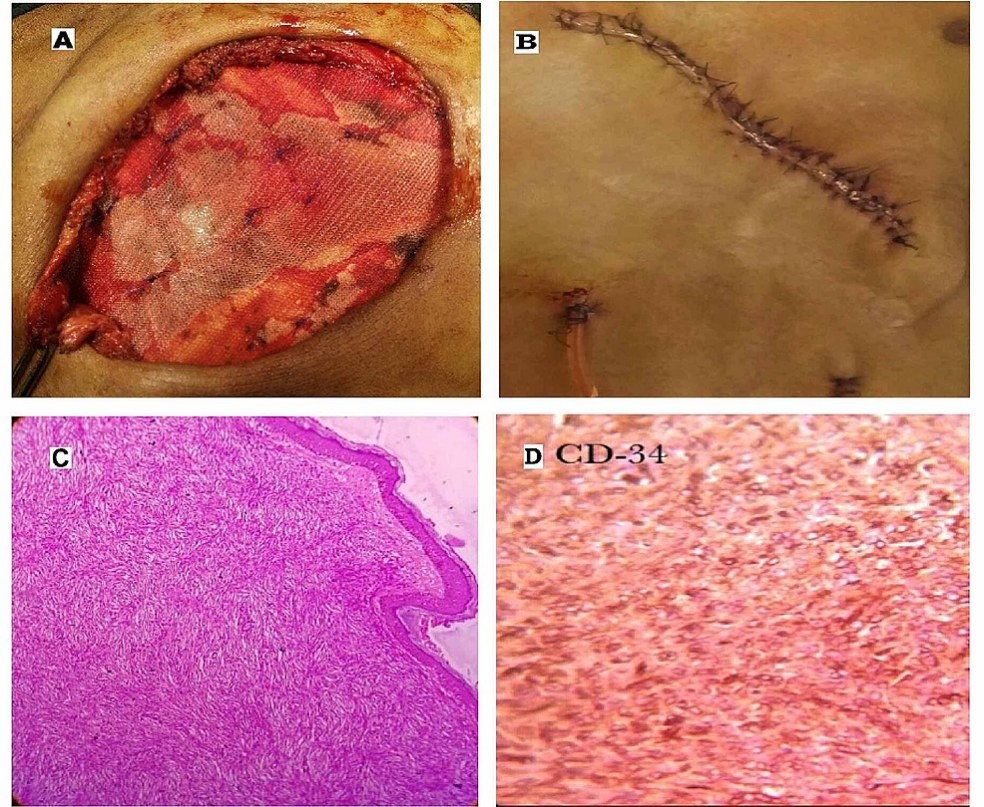
Defect in abdominal wall post excision confirmatory microscopic view. 2(A): Reconstruction of the anterior abdominal wall done by suturing polypropylene mesh to defect margin preperitoneally. 2(B): Full-thickness abdominal wall reconstruction with skin flap approximation done by anterior component separation over the bridging mesh for functional and aesthetic repair. 2(C): The histopathological section showing spindle-shaped tumor cells in the dermis arranged in a storiform pattern. Free Grenz zone with the normal epidermis, along with infiltration with tumor cells into the subcutaneous tissue (HE staining 40X). 2(D): Immunohistochemistry of tumor cells showing cluster of differentiation for CD 34 antigen (magnification 40X).

After primary peritoneal closure, a preperitoneal polypropylene mesh was sutured to the defect margin of the posterior rectus sheath as a bridging mesh for a tension-free repair (Figure 2A). Then, by anterior CST, a myofascial flap of anterior abdominal wall muscles from both sides was approximated medially over the mesh along the oblique incision line to fill the defect, and the abdomen closed in a single layer with suction drainage (Figure 2B). Microscopically, monotonous, spindle-shaped tumor cells arranged in storiform patterns with well-differentiated fat cells entrapped within the spindle cells were seen in the subcutaneous layer with a superﬁcial Grenz zone separating the normal epidermis (Figure 2C). All the margins were free of tumor cells. Ki67 was focally positive in 2-4% of tumor cells, but areas of sarcomatous transformation were not found. Immunohistochemistry (IHC) showed strongly positive CD34 and vimentin and negative for smooth muscle actin (SMA) and S-100, confirming the diagnosis of DFSP (Figure 2D). Post-operative recovery was uneventful. No local recurrence or metastases were found during regular follow-up for the last five years.

## Discussion

DFSP is a superficial fibroblastic dermal sarcoma of intermediate malignancy. The pathogenesis is still uncertain but 10-20% have a history of trauma and in 80-90% of cases, there is somatic mutation by a proto-oncogene COL1A1-PDGFB. This is formed by translocation of 17 and 22 chromosomes, which causes activation of the tyrosine-kinase pathway leading to PDGFβ overexpression [4].

DFSP mostly presents as a slow-growing, painless, superficial, lobulated, and protuberant tumor over the trunk (40-50%) among the 20-50 years age group. The overlying skin is thin, shiny, telangiectatic, and adherent to the tumor [2]. Radio imaging (CT and MRI) is useful for preoperative evaluation, surgical planning, and diagnosis of distant metastasis and recurrence [3,5].

Distinctive histopathological findings of DFSP are spindle-shaped neoplastic cells arranged in a storiform or cartwheel pattern in the dermis, with neoplastic tentacles extending into the surrounding subcutaneous tissues [5]. Hence, tumor resection with a negative margin is difficult, resulting in high local recurrence. Immunohistochemical staining, showing strong positivity of CD34 and vimentin and negative S-100, is diagnostic of DFSP [6,7]. Sarcomatous changes occurring in 10-15% of DFSP, are associated with a giant size and poor prognosis in comparison to classical DFSP [6]. Our case is a giant classical DFSP but without fibrosarcomatous changes, which is very rare.

Very few studies are reported on the management of abdominal wall DFSP. Complete surgical excision with histologically negative margins is the standard treatment of DFSP [5]. This can be achieved by WLE or Mohs micrographic surgery depending on the size, location of the tumor, and availability of expertise [5]. As per National Comprehensive Cancer Network (NCCN) guidelines (version 1.2020), the standard recommendations for improving recurrence-free survival is complete three-dimensional WLE of the tumor with 2-4 cm negative histological margin along with skin, surrounding tissue, and underlying deep fascia [3,5]. In truncal DFSP and resource-challenged centers, WLE is preferred because of easy expertise availability, less time-consuming, cost-effective, and in a single stage, excision with reconstruction can be done with excellent cosmetic and functional outcomes [2,5,8]. The absence of neoplastic cells on margins is the most important prognostic factor for local recurrence [7]. In this case, microscopic negative margins were ensured by three-dimensional excision with three centimeters wide surgical margin, peri-operative frozen section margin evaluation, and an additional shaved margin for histopathological evaluation. This protocol of excision of an additional 1 cm to the standard 3 cm margin in WLE of huge (>5cm) DFSP along with immediate reconstruction after negative frozen section resection margins, has been done by Kim BJ in Korea with very few recurrences [7].

After WLE, the reconstruction of the resultant full-thickness defect aims to protect the abdominal viscera, restoring the functional integrity of the abdominal wall along with its aesthetic appearance and hernia prevention [9,10]. Reconstruction of Type II and III large abdominal wall defects (> 6 cm in diameter) is generally done by vascularized autologous free or pedicled flaps that need the expertise of plastic surgeons [9].

AWR by CST is a recently developed technique of ventral hernioplasty with the principle of re-establishing a functional abdominal wall with autologous tissue repair. This technique can be modified and used in the reconstruction of post-oncologic full-thickness abdominal wall defects after abdominal DFSP excision [9,10]. So primary repair of full-thickness abdominal wall defect of Type III C, after WLE for DFSP, can be achieved by CST as it minimizes the defect size by the advancement of musculofascial layer over the bridging mesh used to cover the defect [9,11]. Advances in minimal invasive CST techniques, such as perforator sparing anterior CST, endoscopic CST, minimally invasive posterior CST, help in decreasing the postoperative wound complications [11]. But this technique is of limited use in the closure of large defects of Type C, of size more than 40 cm2 with wide loss of domain, wherein free musculo fasciocutaneous flaps or vascularized pedicled flaps are preferred [9].

In the reported case, the resultant abdominal wall defect was repaired by AWR with anterior CST and bridge meshplasty for a single staged, tension-free primary functional reconstruction, customized as per the position of the defect. This technique is functional, cost-effective, avoids multiple surgeries, and can be done in a resource-limited setting even by experienced general surgeons. The aim was to simplify the surgical approach, decrease the operative time, cost, morbidity of multiple surgeries, and thus infection rate. So by the preoperative marking of tumor extent with MRI, WLE with 3 cm clear lateral and deep margins, preoperative frozen section histology along with extra shaved margins, aids in achieving oncological negative margins resection. A functional reconstruction can be achieved by AWR by anterior CST and meshplasty.

In recurrent lesions or positive margins, re-excision followed by adjuvant radiotherapy or targeted therapy with tyrosine kinase inhibitors like imatinib is advocated [7]. The prognosis of DFSP after adequate surgical excisions is very good. A longer duration of follow-up, over five years, has been associated with decreased morbidity and mortality [2]. Recurrences are seen mostly within three years but may occur later, so long-term follow-up is mandatory at an interval of 6-12 months [5].

## Conclusions

Awareness of DFSP, a rare surgically curable but locally aggressive dermal sarcoma, is important for a proper diagnosis and management to prevent recurrences. An integrated approach, involving surgery, histopathology, and immuno-radiotherapy, can act as an elixir for making DFSP a curative entity. Anterior CST with meshplasty is an accepted hernia repair technique that can be used for immediate AWR after oncological resection of DFSP with good results by general surgeons in resource-limited places. So to improve the functional and aesthetic outcome without compromising on oncological safety and hernia prevention, a modified three-dimensional excision of the tumor with deep fascia and 3 cm circumferential safe surgical margin, confirmed by frozen section with immediate reconstruction by CST with mesh reinforcement is recommended by us in full-thickness abdominal wall defects following DFSP resection.
